# Total Failure of Fenbendazole to Control Strongylid Infections in Czech Horse Operations

**DOI:** 10.3389/fvets.2022.833204

**Published:** 2022-02-21

**Authors:** Jana Nápravníková, Marián Várady, Jaroslav Vadlejch

**Affiliations:** ^1^Department of Zoology and Fisheries, Czech University of Life Sciences Prague, Prague, Czechia; ^2^Institute of Parasitology, Slovak Academy of Sciences, Košice, Slovakia

**Keywords:** equine strongyles, anthelmintic resistance, fecal egg count reduction test, Mini-FLOTAC, anthelmintic drug

## Abstract

The control of strongylid infections has become challenging globally for equine practitioners due to the development of anthelmintic resistance. Comprehensive information on anthelmintic resistance in the Czech Republic, however, is still lacking. This study monitored the current efficacy of fenbendazole, pyrantel embonate, ivermectin and moxidectin. Forty-eight of 71 operations met the criteria (≥6 horses with ≥200 eggs per gram), with 969 fecal egg count reduction tests performed. Anthelmintic resistance was evaluated on an operation level based on fecal egg count reduction (FECR) and the lower limit of the 95% credible interval (LLCI) using Bayesian hierarchical models. General anthelmintic efficacy across all operations was assessed by posterior FECRs and the occurrence of sub-zero efficacies. Ivermectin and moxidectin demonstrated excellent efficacy (FECR 99.8–100%; 99.4–100 LLCI) in 45 and 23 operations, respectively, pyrantel embonate demonstrated sufficient efficacy in 15 operations and resistance was suspected in seven operations (FECR 88.1–99.1%; 72.5–98.5 LLCI). Fenbendazole, however, was not effective in a single operation (FECR 19.1–77.8%; 8.1–50.1 LLCI) out of 18. Fenbendazole had the highest probability of sub-zero efficacy (29.1%), i.e., post-treatment fecal egg counts exceeded the pre-treatment counts. Our data indicate an increase in the development of anthelmintic resistance, resulting in total failure of fenbendazole and a reduced efficacy of pyrantel embonate. Introducing advanced approaches of parasite control in the Czech Republic to slow the spread of anthelmintic resistance is thus needed.

## Introduction

Strongylid nematodes, particularly cyathostomins, are ubiquitous in equine operations and are currently considered to be the main equine parasites at risk of developing anthelmintic resistance (AR) and causing associated health consequences (1, 2). The spread of AR is in the spotlight for both parasitologists and equine practitioners around the world. Strongylid resistance has been recorded for all equine anthelmintics currently used (3), so the control of these infections has become challenging.

AR is characterized by the genetically transmitted loss of sensitivity to a formerly effective drug in the parasite population at the dose recommended by the manufacturer. The development of AR is based on the selection of specific alleles under drug pressure (4). Fenbendazole (FBZ) resistance is currently the rule rather than the exception in Europe (5–12), the USA (13–15), Chile (16), Cuba (17) and Brazil (18, 19). Resistance to pyrantel (PYR) has progressively spread (4, 7–11, 13, 18, 20–23), but macrocyclic lactones (MLs) usually maintain sufficient efficacy. Early signs of resistance to MLs, such as shortened periods of egg reappearance (24–26) or fully developed AR confirmed by fecal egg count reduction tests (FECRTs), however, have been reported (8, 11, 18).

Four anthelmintics belonging to three classes based on chemical structure and pharmacological behavior are used for controlling strongylid infections in the Czech Republic: FBZ, a benzimidazole (BZ); pyrantel embonate (PYR), a tetrahydropyrimidine and two MLs, ivermectin (IVM), an avermectin, and moxidectin (MOX), a milbemycin. Limited data on the resistance of strongylids to these drugs, however, are available. Several AR studies have been conducted in the Czech Republic but were local studies and small-scale studies. No nationwide studies evaluating all anthelmintics registered for the control of strongylid infections have yet been performed. BZ resistance and sufficient IVM efficacy have been reported (27–29), but limited data on PYR (30) resistance and no data on MOX efficacy in the Czech Republic are available.

In the first nationwide study, we evaluated the efficacy of all anthelmintics currently used in the Czech Republic to control strongylid infections in horses.

## Materials and Methods

### Equine Operations

**Figure 1:**
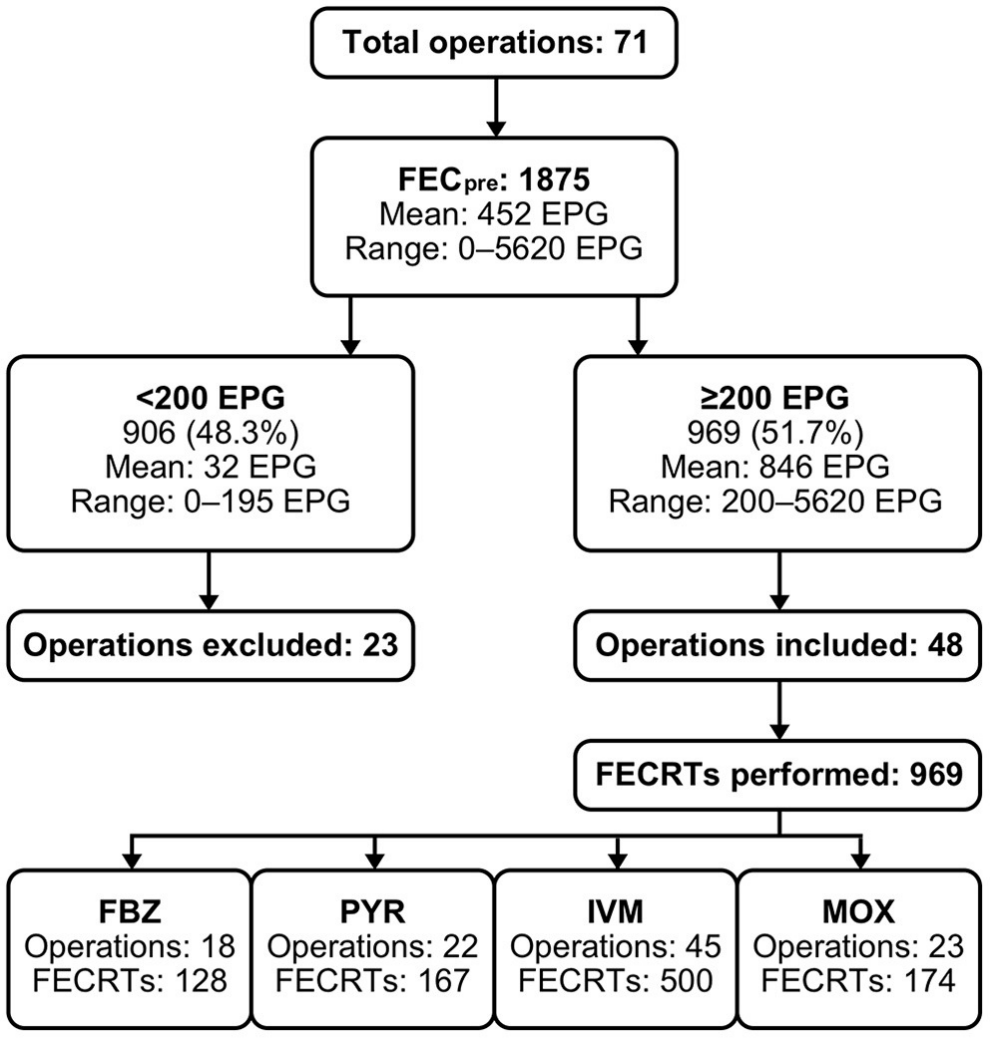
Flow diagram of the study design and FEC_pre_ results represented by EPG prior to the statistical analysis of the FECRTs for FBZ, PYR, IVM and MOX. EPG, eggs per gram; FBZ, fenbendazole; FEC_pre_, initial pre-treatment fecal egg count; FECRT, fecal egg count reduction test; IVM, ivermectin; MOX, moxidectin; PYR, pyrantel embonate.

The data presented in this study were obtained in 2019 and 2020 from equine operations across the Czech Republic. Out of a total of 71 operations, 48 met the criteria for resistance testing (2–4 operations in each of the 14 regions). Mares, geldings and stallions aged 2–28 years with diverse functions (stud, show, leisure, therapy) were included. Operations followed a routine plan of treatment frequency (2–4/year) and anthelmintic choice determined by local veterinary practitioners. Only operations with a minimum of a 16-week period since the previous treatment were included. The flow diagram in Figure 1 shows the design of the study and the selection of operations based on our criteria.

### Parasitological Procedures

The efficacies of FBZ, PYR, IVM and MOX were estimated using the fecal egg count reduction test (FECRT), the estimation of anthelmintic efficacy *via* post-treatment egg reduction (31–33). The Mini-FLOTAC technique (34) with the Fill-FLOTAC (35) apparatus was used following the protocol recommended for fresh herbivore feces (5 g feces; 45 ml flotation solution at a specific gravity of 1.28; multiplication factor of 5). This technique is based on the passive flotation of eggs in flotation chambers with total volumes of 2 ml and is characterized by a revolving reading disc that provides improved readability. The disk were examined by an experienced technician using an Olympus BX51 microscope at a magnification of 100×. The eggs were then morphologically identified (36).

Initial fecal egg counts (FEC_pre_) for all horses from each operation were performed to determine whether the operation met the criteria for the FECRT, i.e., a minimum of six horses with ≥200 eggs per gram (EPG). Operations that did not fulfill these requirements were excluded. A subsequent fecal egg count (FEC_post_) followed 14 days after anthelmintic application and was performed in a selected group of horses (≥200 EPG). Individual fecal samples were collected immediately after defecation, placed into airtight zip-lock bags, transported to the laboratory, refrigerated (4°C) and processed within 24 h after collection.

### Anthelmintic Treatment

Registered anthelmintics available in the Czech Republic (FBZ: Panacur, Intervet International, Boxmeer, Netherlands; PYR: EQUISTRONG, Bioveta, Ivanovice na Hané, Czech Republic; IVM: NOROMECTIN, Norbrook Laboratories, Monaghan, Ireland; MOX: EQUIMOXIN, Bioveta, Ivanovice na Hané, Czech Republic) were administered per os by an authorized person in a single dose recommended by the manufacturer (FBZ 7.5 mg/kg body weight (BW); PYR 19 mg/kg BW; IVM 0.2 mg/kg BW; MOX 0.4 mg/kg BW) based on estimates of body weight (tape measurements). The expiry dates were checked before application. The number of anthelmintics tested in one operation varied depending on the total number of horses in the operation, the number of horses with sufficient FECs and the common local practices such as the number of anthelmintic treatments per year.

### Statistical Analyses

The FECRs for individual horses were calculated by a Bayesian hierarchical model analysis of the data using an estimate of mean FECR and 95% credible intervals (CIs) (37, 38). FECR (%) was calculated for each horse, and mean FECRs, 95% CIs and the means and ranges of FEC_pre_ and FEC_post_ were calculated for each operation. Data representing the anthelmintic efficacy in horses and particular operations are displayed in the tables and column graphs attached as Supplementary Material (unabridged tables).

**Figure 2:**
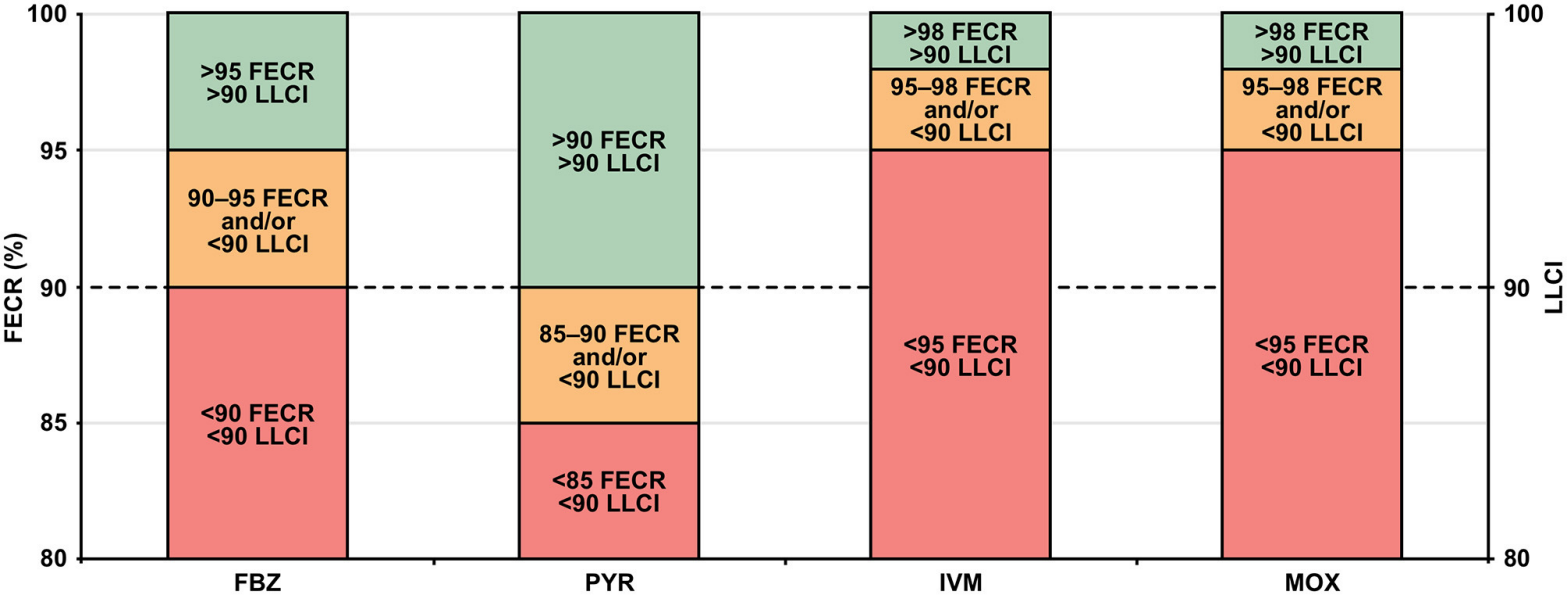
Criteria for determining the efficacies of FBZ, PYR, IVM and MOX: FECR% (normal, green; suspected, orange; reduced, red) and 95% LLCI. FBZ, fenbendazole; FECR%, mean fecal egg count reduction; IVM, ivermectin; 95% LLCI, lower limit of the 95% credible interval; MOX, moxidectin; PYR, pyrantel embonate.

Drug efficacy (normal, suspected and reduced) at the operation level was determined using mean FECR (%) and the lower limit of the 95% CIs (LLCI) (Figure 2) (33, 39).

General efficacy was also calculated for each anthelmintic regardless of affiliations with individual operations (subsamples) and is graphed as the posterior distribution of FECR. An analysis of sub-zero efficacies (FECR <0%) with confidence intervals in individual horses was performed with restriction efficacies to interval 0 to 1 and random variabilities between operations.

## Results

FEC_pre_ was performed in 71 operations and 1,875 horses, of which 48.3% can be considered low (0–195 EPG), 19.7% moderate (200–500 EPG), and 32.0% high (>505 EPG) contaminators. Horses with 0 EPG represented 23.3% of the total.

Twenty-three operations were excluded from resistance testing for not meeting the condition of the sufficient number of horses with sufficient FEC_pre_. Therefore, 969 FECRTs were performed in the remaining 48 operations (Figure 1 and Table 1).

**Table 1:** Estimated efficacies of fenbendazole (FBZ), pyrantel embonate (PYR), ivermectin (IVM) and moxidectin (MOX) at the operation and horse levels.

**Efficacy**	***n***		**Reduced**		**Suspected**		**Normal**	
	**Operations**	**Horses**	**Operations**	**Horses**	**Operations**	**Horses**	**Operations**	**Horses**
FBZ	18	128	18 (100%)	122 (95.3%)	–	1 (0.8%)	–	5 (3.9%)
PYR	22	167	–	10 (6.0%)	7 (31.8%)	3 (1.8%)	15 (68.2%)	154 (92.2%)
IVM	45	500	–	–	–	–	45 (100%)	500 (100%)
MOX	23	174	–	–	–	–	23 (100%)	174 (100%)

### Operation-Level Efficacy

**Figure 3:**
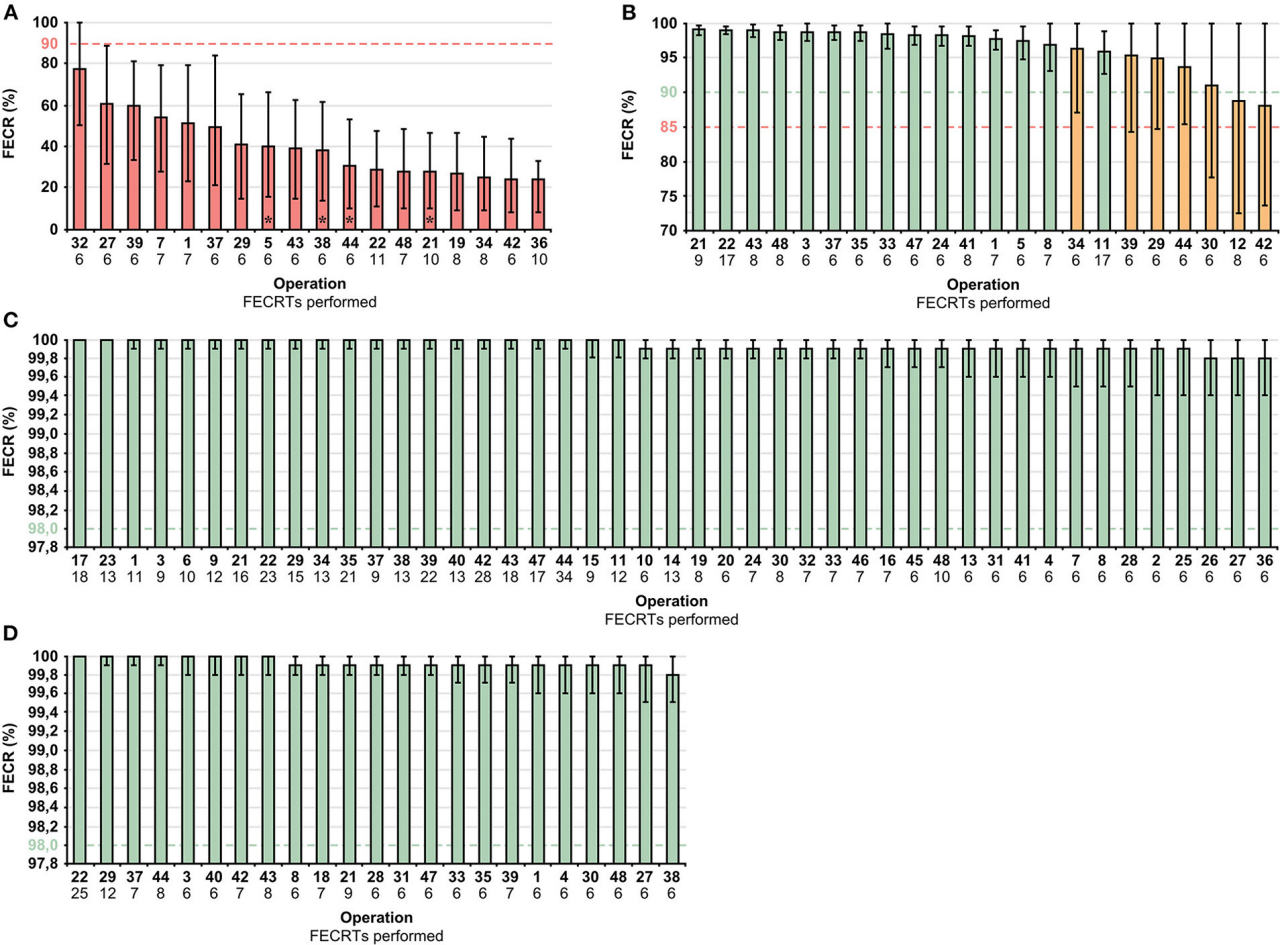
Estimated efficacies of FBZ **(A)**, PYR **(B)**, IVM **(C)** and MOX **(D)** (normal, green; suspected, orange; reduced, red) based on FECR% and 95% LLCI at the operation level. The dashed lines represent the criteria for estimating FECR% efficacy for each anthelmintic, error bars 95% CI (credible interval), *sub-zero efficacy at operation level (mean FECRT <0%). FBZ, fenbendazole; FECR%, mean fecal egg count reduction; IVM, ivermectin; MOX, moxidectin; PYR, pyrantel embonate; 95% LLCI, lower limit of 95% credible interval.

The estimates of efficacy of the four anthelmintics in individual equine operations were visualized using column graphs (Figure 3) with cut-off values (horizontal dashed line) highlighted. The columns represent mean FECR (%) and are arranged in descending order for clarity. Unabridged data are attached in the Supplementary Material.

FBZ demonstrated reduced efficacy (mean FECR 19.1–77.8%; 8.1–50.1 LLCI) in all operations, PYR predominantly demonstrated normal efficacy with suspected resistance in seven operations (mean FECR 88.1–99.1%; 72.5–98.5 LLCI) and IVM (mean FECR 99.8–100%; 99.4–100 LLCI) and MOX (mean FECR 99.8–100%; 99.5–100 LLCI) demonstrated normal efficacies in all operations. Dual-drug resistance (FBZ resistance and suspected PYR resistance) was suspected in five operations (21, 22, 37, 43 and 48).

### General Efficacy

**Figure 4:**
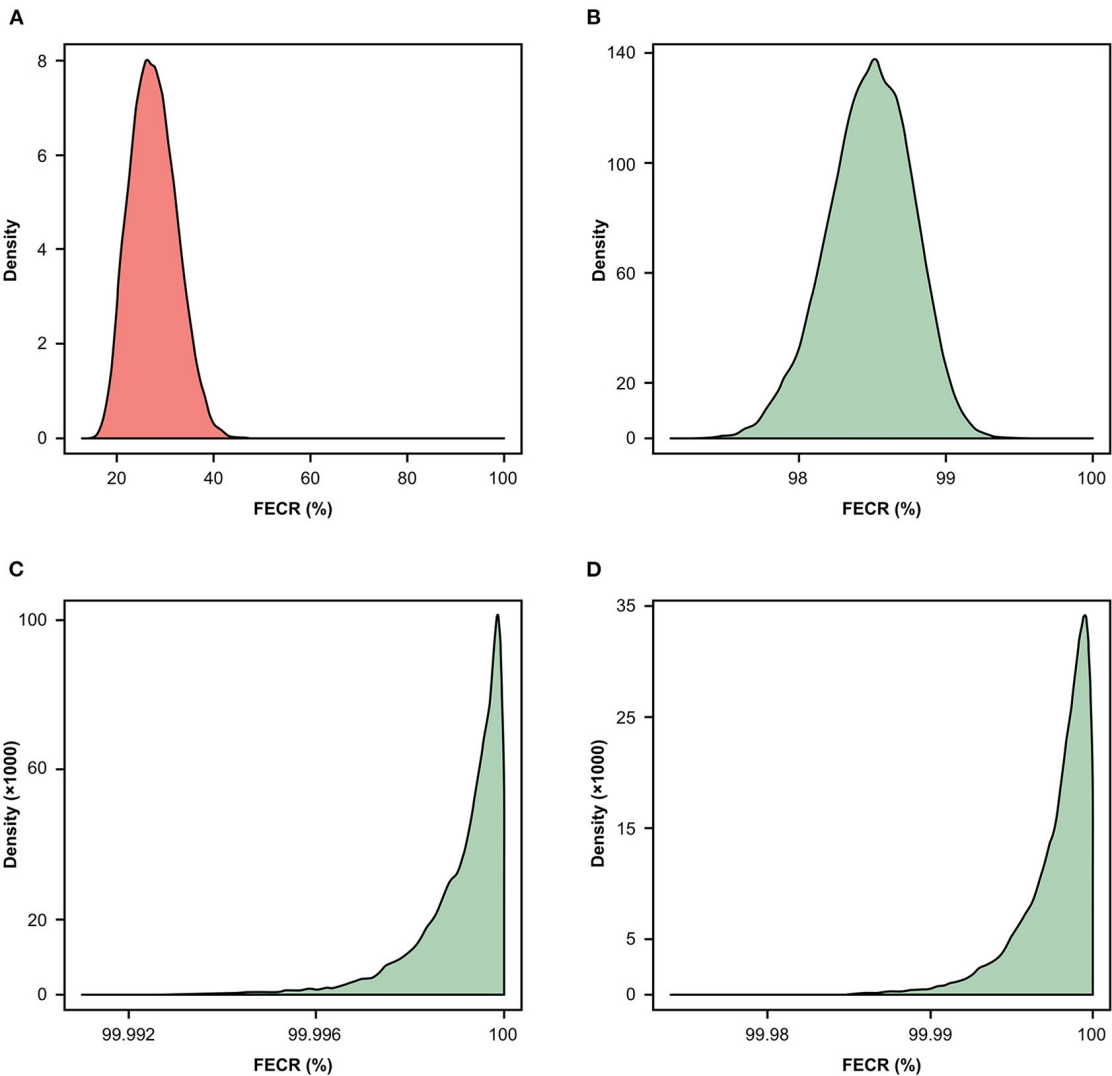
Posterior probability distribution of FECR% for FBZ **(A)**, PYR **(B)**, IVM **(C)** and MOX **(D)** at the horse level. FBZ, fenbendazole; FECR%, fecal egg count reduction; IVM, ivermectin; MOX, moxidectin; PYR, pyrantel embonate.

Figure 4 presents the general efficacies of the anthelmintics visualized as the posterior distribution of fecal egg reduction. Almost one-third (29.1%; CI 21.9–37.6) of the FBZ treatments resulted in sub-zero efficacies (individual FEC_post_ exceeding FEC_pre_), and the occurrence of sub-zero efficacies was minimal for PYR (0.6%; CI 0.1–4.2) and absent for the MLs. The probability of occurrence of sub-zero efficacies for FBZ and PYR compared to the MLs differed significantly (*p* < 0.0001) among the horses.

## Discussion

The equine industry in the Czech Republic is growing, with the number of registered equines in 2021 exceeding the 100,000 mark for the first time. This study provides the first comprehensive data on the efficacy of all anthelmintic compounds used in Czech horse operations. All equine anthelmintics in the Czech Republic are available only with a prescription, and their distribution strictly relies on veterinary practitioners. Individual horses maintain their shedding potential, and the majority of eggs are produced by a small portion of herd individuals (40, 41), but the strategic approach lacking adequate measures to determine the need to administer anthelmintics and verify the efficacy to avoid ineffective drugs is still commonly practiced in the Czech Republic. Nearly half of the horses in our study were considered low contaminators, because they shed fewer than 200 EPG. Most of the horses, however, still received treatment using the customary approach to treat all horses at fixed times of the year. In contrast, treatment twice a year could be insufficient for high shedders to avoid the excessive contamination of pastures. The threshold for the selective-treatment approach has not been precisely determined for horses and could vary depending on individual conditions. Horses classified as moderate (200–500 EPG) and high (>500 EPG) contaminators generally shed the majority of eggs and require anthelmintic treatment (28).

A high level of resistance was confirmed for FBZ, resistance was suspected for PYR due to the sporadic incidence of reduced efficacy in individual horses in an operation and MLs remained fully effective.

Our results suggest that although MLs are currently used the most frequently (IVM 42%, MOX 21%), compared to PYR (20%) and FBZ (17%), they remain highly effective, and the development of AR is slow. The importation of horses and ineffective quarantine measures, however, can strongly affect the spread of ML-resistant strongylids (42). Reports of resistance to MLs in Europe (8, 11, 33, 43) indicate the potential risk of ML-resistant strongylids. Our results nevertheless indicated excellent efficacy for the MLs and confirmed the results of previous studies (27–29). These results may be due to the uncommon use of the interval strategy in the Czech Republic. The overuse of anthelmintics and the minimal use of refugia are known factors for the development of AR (1).

We did not detect fully developed resistance to PYR, but 32% (7 of 22) of the operations demonstrated suspected resistance, because some horses had decreased FECRs (8% of the horses had FECRs <90%). The data obtained from our study highlight the need to verify the efficacy of regular PYR treatment (32) for the early detection of AR. FBZ resistance is ubiquitous, so potential multidrug resistance can lead to the exclusive use of MLs associated with the risk of accelerating the emergence of resistance.

BZ-resistant strongylids are pervasive in the Czech Republic. The value of the FECRT continues to decrease over time, and the incidence of individual sub-zero efficacies is increasing compared to previous studies (27–30). Avoiding the use of FBZ to control strongylid infections is essential for preventing economic and health consequences. FBZ was introduced to the Czech market in 1976, and IVM was introduced 10 years later (personal communication). Both anthelmintics were used under similar conditions, but FBZ lacks efficacy and IVM remains fully effective. Other factors possibly affecting AR need to be considered. Product formulation and the size of the packaging may indirectly influence the exact dosage. Powder and granules (FBZ) mixed with grain are not willingly accepted by all horses, and repeated underdosing may occur (44). The dose of a drug in an applicator insufficient for standard warm-blooded animals (e.g., 450 kg BW mebendazole, a BZ) could tempt horse owners, due to economic reasons, to administer only one paste to a horse requiring a larger amount. Finally, the variety of concurrently marketed products of the same anthelmintic class (e.g., FBZ and mebendazole) could substantially increase the use of one anthelmintic class with a false impression of rotation of anthelmintics with different modes of action (45).

Various approaches for analyzing anthelmintic efficacy make the comparison of the results among studies challenging. FECRT is currently a gold standard in AR detection, but it still has limitations such as low sensitivity, variable reliability of the coprological FEC methods used (46), the lack of standardization and cut-off values for horses and the difficulty of interpretation. The high species diversity of equine strongylids is also an important factor.

In conclusion, this study provides comprehensive information about the current situation of the resistance of equine strongylids to anthelmintics in the Czech Republic. FBZ is no longer effective for strongylid control. PYR resistance was suspected in some operations and should therefore be used with caution due to the potential risk of the development dual resistance. In contrast, the MLs still had sufficient efficacy, which must be maintained as long as possible for detecting early signs of AR (e.g., period of egg reappearance). Modern approaches to strongylid control, e.g., non-chemical approaches or selective anthelmintic treatment, need to be implemented but will require educating both horse owners and veterinarians. Identifying the risk factors that accelerate the development of AR, and further research oriented in this direction, are essential.

## Data Availability Statement

The original contributions presented in the study are included in the article/Supplementary Material, further inquiries can be directed to the corresponding author.

## Ethics Statement

Ethical review and approval was not required for the animal study because this study involved the examination of naturally excreted fecal samples. Written informed consent was obtained from the owners for the participation of their animals in this study.

## Author Contributions

JN designed the study, collected the samples, performed the laboratory work, analyzed the data, interpreted the results, and prepared the manuscript. MV interpreted the results and reviewed the manuscript. JV designed the study, supervised the project, and reviewed the manuscript. All authors contributed to the article and approved the submitted version.

## Funding

This research was partially supported by the Ministry of Education, Youth and Sports INTER-EXCELLENCE project (INTER-COST LTC19018) and was performed within the initiative of the Center for Infectious Animal Diseases (CINeZ).

## Conflict of Interest

The authors declare that the research was conducted in the absence of any commercial or financial relationships that could be construed as a potential conflict of interest.

## Publisher's Note

All claims expressed in this article are solely those of the authors and do not necessarily represent those of their affiliated organizations, or those of the publisher, the editors and the reviewers. Any product that may be evaluated in this article, or claim that may be made by its manufacturer, is not guaranteed or endorsed by the publisher.
