# Supplementary material for: Total Failure of Fenbendazole to Control Strongylid Infections in Czech Horse Operations
Source: Front Vet Sci. 2022 Feb 21;9:833204. doi: 10.3389/fvets.2022.833204 (PMC8899116; doi:10.3389/fvets.2022.833204)
Supplement: Supplementary file 1 [file Data_Sheet_1.PDF]

Supplementary Material

Supplementary Table 1. Unabridged data for estimates of fenbendazole (FBZ) efficacy at the operation level calculated using fecal egg count (FEC) and arranged in descending order of fecal egg count reduction (FECR).

FBZ Operation	n	FEC_{pre} (EPG)		FEC_{post} (EPG)		FECR (95% CI)
		Mean	Range	Mean	Range	
32	6	933	240–1725	1213	0–3870	77.8% (50.1–100)
27	6	476	250–890	218	0–585	60.6% (31.2–89.2)
39	6	741	200–1710	331	10–825	59.6% (33.3–81.5)
7	7	1271	300–3735	427	80–1305	53.8% (27.9–80.0)
1	7	829	370–1300	532	0–1045	51.5% (23.5–79.5)
37	6	782	220–1530	988	435–2190	49.6% (21.0–83.9)
29	6	533	240–1230	408	35–965	40.9% (14.5–65.1)
5	6	1112	595–1990	836	35–2115	40.1% (15.4–66.0)
43	6	954	320–2035	638	205–1475	39.8% (14.5–62.4)
38	6	645	225–1110	384	180–665	38.6% (13.8–62.0)
44	6	650	325–995	577	185–1290	31.0% (10.4–53.3)
22	11	1429	405–3855	975	200–2560	29.0% (11.2–48.0)
48	7	656	260–1735	536	170–1140	28.2% (9.8–49.2)
21	10	636	210–1285	531	100–915	27.5% (9.9–46.4)
19	8	759	240–1265	638	255–910	27.2% (9.3–46.8)
34	8	702	315–2235	659	175–2040	25.4% (9.3–44.7)
42	6	788	250–1955	842	195–1900	24.6% (8.7–44.2)
36	10	885	375–1550	976	410–2100	19.1% (8.1–33.4)

CI, credible interval; EPG, eggs per gram; FEC_{post}, post-treatment fecal egg count; FEC_{pre}, pre-treatment fecal egg count.

Supplementary Table 2. Unabridged data for estimates of pyrantel embonate (PYR) efficacy at the operation level calculated using fecal egg count (FEC) and arranged in descending order of fecal egg count reduction (FECR).

PYR Operation	n	FEC_{pre} (EPG)		FEC_{post} (EPG)		FECR (95% CI)
		Mean	Range	Mean	Range	
21	9	718	220–2010	6	0–20	99.1% (98.3–99.7)
22	17	674	210–2020	7	0–30	99.0% (98.5–99.6)
43	8	619	240–1145	6	0–20	99.0% (98.0–99.8)
48	8	484	255–935	5	0–15	98.8% (97.6–99.7)
3	6	553	205–1145	5	0–15	98.8% (97.5–99.9)
37	6	871	255–2220	10	0–25	98.7% (97.6–99.7)
35	6	658	210–1855	9	0–40	98.7% (97.4–99.7)
33	6	541	410–810	7	0–20	98.4% (96.3–100)
47	6	584	205–935	9	0–20	98.3% (96.9–99.5)
24	6	543	300–950	9	0–35	98.3% (96.7–99.6)
41	8	696	365–1085	10	0–20	98.2% (96.7–99.5)
1	7	1294	250–3010	34	0–100	97.8% (96.2–99.0)
5	6	1124	590–2115	20	0–35	97.4% (94.8–99.5)
8	7	787	270–1525	19	0–45	96.8% (97.7–100)
34	6	608	460–955	28	0–150	96.4% (87.1–100)
11	17	782	275–1260	48	0–385	95.9% (92.6–98.9)
39	6	653	205–1310	49	0–265	95.3% (84.2–100)
29	6	798	255–1880	56	0–270	95.0% (84.7–100)
44	6	553	250–725	38	0–115	93.6% (85.4–100)
30	6	673	200–1145	57	0–300	91.0% (77.7–100)
12	8	814	215–2035	81	0–315	88.8% (72.5–100)
42	6	1052	245–2170	150	0–600	88.1% (73.6–100)

CI, credible interval; EPG, eggs per gram; FEC_{post}, post-treatment fecal egg count; FEC_{pre}, pre-treatment fecal egg count.

Supplementary Table 3. Unabridged data for the estimates of ivermectin (IVM) efficacy at the operation level calculated using fecal egg count (FEC) and arranged in descending order of fecal egg count reduction (FECR).

IVM Operation	n	FEC_{pre} (EPG)		FEC_{post} (EPG)		FECR (95% CI)
		Mean	Range	Mean	Range	
17	18	1691	335–3780	0	–	100% (100–100)
23	13	1485	295–4635	0	–	100% (100–100)
1	11	825	375–1800	0	–	100% (99.9–100)
3	9	969	200–2150	0	–	100% (99.9–100)
6	10	827	200–2230	0	–	100% (99.9–100)
9	12	804	220–2040	0	–	100% (99.9–100)
21	16	1028	295–2470	0	–	100% (99.9–100)
22	23	720	200–1650	0	–	100% (99.9–100)
29	15	1223	330–2935	0	–	100% (99.9–100)
34	13	1278	245–3150	0	–	100% (99.9–100)
35	21	798	210–1465	0	–	100% (99.9–100)
37	9	1391	215–4850	0	–	100% (99.9–100)
38	13	526	210–1540	0	–	100% (99.9–100)
39	22	822	200–1875	0	–	100% (99.9–100)
40	13	649	200–2630	0	–	100% (99.9–100)
42	28	836	200–4110	0	–	100% (99.9–100)
43	18	524	220–1050	0	–	100% (99.9–100)
47	17	749	250–2140	0	–	100% (99.9–100)
44	34	756	205–2525	0	–	100% (99.9–100)
15	9	853	245–1300	0	–	100% (99.8–100)
11	12	710	225–2480	0	–	100% (99.8–100)
10	6	1085	675–2235	0	–	99.9% (99.8–100)
14	13	466	215–1025	0	–	99.9% (99.8–100)
19	8	638	255–910	0	–	99.9% (99.8–100)
20	6	1292	285–3440	0	–	99.9% (99.8–100)
24	7	838	315–1760	0	–	99.9% (99.8–100)
30	8	869	225–3850	0	–	99.9% (99.8–100)
32	7	921	220–3725	0	–	99.9% (99.8–100)
33	7	845	320–2215	0	–	99.9% (99.8–100)
46	7	868	240–1865	0	–	99.9% (99.8–100)
16	7	756	250–1295	0	–	99.9% (99.7–100)
45	6	856	275–1375	0	–	99.9% (99.7–100)
48	10	477	275–710	0	–	99.9% (99.7–100)

13	6	677	385–1220	0	–	99.9% (99.6–100)
31	6	653	270–1135	0	–	99.9% (99.6–100)
41	6	748	205–1120	0	–	99.9% (99.6–100)
4	6	715	200–1375	0	–	99.9% (99.6–100)
7	6	582	220–1305	0	–	99.9% (99.5–100)
8	6	548	470–755	0	–	99.9% (99.5–100)
28	6	535	215–885	0	–	99.9% (99.5–100)
2	6	464	200–740	0	–	99.9% (99.4–100)
25	6	503	220–1335	0	–	99.8% (99.4–100)
26	6	439	205–1100	0	–	99.8% (99.4–100)
27	6	513	205–1250	0	–	99.8% (99.4–100)
36	6	443	255–720	0	–	99.8% (99.4–100)

CI, credible interval; EPG, eggs per gram; FEC_{post}, post-treatment fecal egg count; FEC_{pre}, pre-treatment fecal egg count.

Supplementary Table 4. Unabridged data for the estimates of moxidectin (MOX) efficacy at the operation level calculated using fecal egg count (FEC) and arranged in descending order of fecal egg count reduction (FECR).

MOX Operation	n	FEC_{pre} (EPG)		FEC_{post} (EPG)		FECR (95% CI)
		Mean	Range	Mean	Range	
22	25	1056	235–3455	0	–	100% (100–100)
29	12	1251	220–2200	0	–	100% (99.9–100)
37	7	1558	720–2990	0	–	100% (99.9–100)
44	8	997	250–1505	0	–	100% (99.9–100)
3	6	1570	505–4900	0	–	100% (99.8–100)
40	6	1417	325–2545	0	–	100% (99.8–100)
42	7	1158	200–2745	0	–	100% (99.8–100)
43	8	944	325–1345	0	–	100% (99.8–100)
8	6	1131	545–1730	0	–	99.9% (99.8–100)
18	7	832	370–1600	0	–	99.9% (99.8–100)
21	9	646	255–1320	0	–	99.9% (99.8–100)
28	6	1155	290–2610	0	–	99.9% (99.8–100)
31	6	790	270–5620	0	–	99.9% (99.8–100)
47	6	1107	380–1850	0	–	99.9% (99.8–100)
33	6	523	255–2135	0	–	99.9% (99.7–100)
35	6	458	275–1950	0	–	99.9% (99.7–100)
39	7	704	265–1100	0	–	99.9% (99.7–100)
1	6	604	230–905	0	–	99.9% (99.6–100)
4	6	695	335–1940	0	–	99.9% (99.6–100)
30	6	323	200–1365	0	–	99.9% (99.6–100)
48	6	678	255–1140	0	–	99.9% (99.6–100)
27	6	493	205–915	0	–	99.9% (99.5–100)
38	6	503	255–995	0	–	99.8% (99.5–100)

CI, credible interval; EPG, eggs per gram; FEC_{post}, post-treatment fecal egg count; FEC_{pre}, pre-treatment fecal egg count.
